# Calibration Transfer Based on Affine Invariance for NIR without Transfer Standards

**DOI:** 10.3390/molecules24091802

**Published:** 2019-05-09

**Authors:** Yuhui Zhao, Ziheng Zhao, Peng Shan, Silong Peng, Jinlong Yu, Shuli Gao

**Affiliations:** 1 School of Computer Science and Engineering, Northeastern University, Shenyang 110819, China; yuhuizhao@neuq.edu.cn (Y.Z.); 13081850350@163.com (Z.Z.); jianren_d@163.com (J.Y.); 15238247216@163.com (S.G.); 2 College of Information Science and Engineering, Northeastern University, Shenyang 110819, China; 3 Institute of Automation, Chinese Academy of Sciences, Beijing 100190, China; silong.peng@ia.ac.cn

**Keywords:** near-infrared (NIR) spectroscopy, calibration transfer, affine invariance, multivariate calibration, partial least squares (PLS)

## Abstract

Calibration transfer is an important field for near-infrared (NIR) spectroscopy in practical applications. However, most transfer methods are constructed with standard samples, which are expensive and difficult to obtain. Taking this problem into account, this paper proposes a calibration transfer method based on affine invariance without transfer standards (CTAI). Our method can be utilized to adjust the difference between two instruments by affine transformation. CTAI firstly establishes a partial least squares (PLS) model of the master instrument to obtain score matrices and predicted values of the two instruments, and then the regression coefficients between each of the score vectors and predicted values are computed for the master instrument and the slave instrument, respectively. Next, angles and biases are calculated between the regression coefficients of the master instrument and the corresponding regression coefficients of the slave instrument, respectively. Finally, by introducing affine transformation, new samples are predicted based on the obtained angles and biases. A comparative study between CTAI and the other five methods was conducted, and the performances of these algorithms were tested with two NIR spectral datasets. The obtained experimental results show clearly that, in general CTAI is more robust and can also achieve the best Root Mean Square Error of test sets (RMSEPs). In addition, the results of statistical difference with the Wilcoxon signed rank test show that CTAI is generally better than the others, and at least statistically the same.

## 1. Introduction

With the characteristics of high efficiency, low cost and non-destructivity, near-infrared (NIR) spectroscopy has been widely used in control of food and pharmaceutical quality [1,2,3,4]. Multivariate calibration methods are commonly used to obtain quantitative or qualitative information from near-infrared spectra, such as principal component regression (PCR) [5,6] and partial least squares (PLS) [7,8,9,10]. Since changes of the instruments and measurement conditions may result in poor applicability of the model. Recalibration can be utilized to solve this problem, but recalibration is time consuming and takes an immense amount of work. In order to reduce consumption of the recalibration, calibration transfer has been widely studied and applied [11]. There are two main situations about calibration transfer: (1) The uniform calibration model is used to predict spectra being measured on multiple instruments; (2) the new spectra are measured on the same instrument after a period of time.

A number of related methods for calibration model transfer have been proposed, which are divided into two categories. Ones require transfer standards and ones not require transfer standards. The first category of methods has the characteristic that a set of samples are separately measured on the master and slave instrument. A great variety of transfer methods with standard samples have been proposed. For examples, SBC [12,13] assumes a linear relationship between predicted values of different instruments. First, the regression coefficient between the spectra and the response values on the master instrument is calculated. Then the predicted values of the master and slave setting are computed based on the regression coefficient. Finally, a linear equation is fitted between the predicted values. PDS proposed by Wang et al. is employed to correct the spectral differences [14]. In PDS [15,16,17,18], each wavelength of the master instrument is related to the wavelength window of the slave instrument, and a band transfer matrix is finally formed based on the regression coefficients of each window. The observation is consistent with this assumption that in various transfer methods the spectral correlation between master and slave is limited to smaller regions. The keys to PDS are the selection of window size and the number of standard samples. Due to the construction of multiple regression models, a huge amount of calculations are desired. The calibration model transfer for near-infrared spectra based on canonical correlation analysis [19] is proposed by Liang et al. The PLS model is built using the master instrument calibration set, and a part of the calibration set of master and slave instrument is taken as standard samples. Then, the features extracted respectively by canonical correlation analysis (CCA) [20,21]. The relationship between master and slave data is established with ordinary least squares (OLS) [22,23], and the test set is finally corrected. For CCA, SBC and PDS, a good result can be achieved with standard samples, but standard samples are difficult to obtain in some cases. For the transfer methods such as calibration transfer via extreme learning machine auto-encoder (TEAM) [24] method, calibration transfer by generalized least squares (GLSW) [25] method and spectral space transform (SST) [26,27] and so on, standard samples are also required, although the principles of these methods are different.

The second category is the methods without transfer standards. For examples, multiplicative scatter correction (MSC) [28,29,30] proposed by Bouveresse et al. first calculates the mean spectra of the calibration set as the reference spectra, then the linear relationship is found between every spectra and the reference spectra, and the slope and bias are obtained; finally, the slope and bias are utilized to correct slave spectra. While the standard samples are not required in MSC, it is difficult to handle complex situations. MSC is a transfer method using pre-processing techniques, and more pre-processing approaches include finite impulse response (FIR) [31] filtering and multivariate filtering via orthogonal signal correction (OSC) [32,33], etc. TCR [34] is also a standard-free method which combines transfer component analysis (TCA) [35] and ordinary least squares (OLS). The basic idea of TCA is to project the data of two instruments in a Reproducing Kernel Hilbert Space, where the data are distributed as close as possible at the same time preserving the key attributes of the original data. TCR is a robust model with good generalization abilities, but does not achieve more accurate predictions. Other techniques belonging to this category include kernel principal component analysis (KPCA) [36,37], domain generalization via invariant feature representation (DICA) [38] and so on.

**Figure 1:**
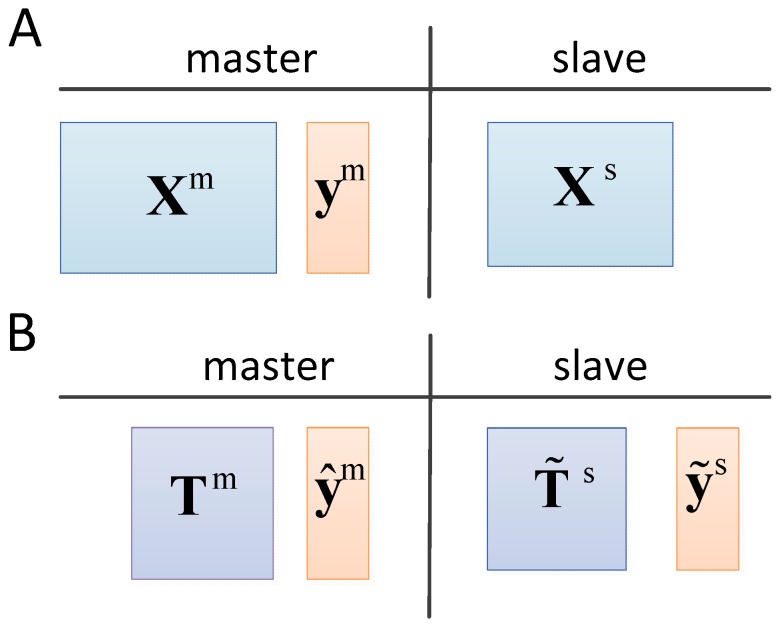
Data setting of the calibration transfer method based on affine invariance without transfer standards (CTAI). We assume the data to be available in (**A**), and the data after being processed based on PLS model of the master instrument is shown in (**B**).

Different from the above methods, this paper studies the relationship of regression coefficients between the feature vector and predicted values on two spectrometers. Samples of the calibration transfer method based on affine invariance without transfer standards (CTAI) are shown in Figure 1A. The response values of the slave spectrometer are not required, and the map is not necessary between master and slave samples. The samples are further processed under the PLS model. The spectral features and prediction values are respectively obtained, and the processed samples are shown in Figure 1B. We obtain the linear models between the feature vector and the predicted values respectively. According to the linear models of two instruments, the relationship between the predicted values is further obtained. Firstly, the PLS model is built on the master instrument; secondly, the score matrices and predicted values are extracted according to the PLS model, respectively; further, the angles and biases are calculated between two regression coefficients; finally, the prediction values are corrected by affine transformation. If the concentration information of the master spectra and the slave spectra are in the same range, CTAI can achieve more accurate predicted results and more robust model even without standard samples compared with other methods. The predictive performance of CTAI is verified by two near-infrared (NIR) datasets.

## 2. Results and Discussion

### 2.1. Analysis of the Corn Dataset

The training errors, prediction errors, cross-validation errors, biases and the correlation coefficients for the predicted vs. actual results about the PLS model of the corn dataset are shown in Table 1. Large correlation coefficients and small biases can be seen in all results. The results reflect a good linear relationship between the spectra and measured values of the corn dataset. There are no significant differences between Root Mean Square Error of calibration set (RMSEC), Minimum Root Mean Square Error of Cross-Validation (RMSECV) and Root Mean Square Error of test set (RMSEP), indicating that there is no over-fitting and under-fitting phenomenon, which can explain the reasonable selection of the number of latent variables. Moreover, we can see that RMSEP^m^ of the PLS on the instrument m5spec are smaller than the RMSEP^m^ of the instrument mp6spec. For most calibration transfer methods, it is important that the master instrument has more accurate prediction results. Thus, m5spec as the master instrument and mp6spec as the slave instrument is a more reasonable choice.

In order to more fully assess the predicted performance of CTAI, the methods MSC, TCR, CCA, SBC and PDS are tested. In this work, when PDS was performed, PLS was utilized to compute the transformation function. For the PLS model, the optimal number of latent variables is shown in Table 1. The optimal dimensionality of the subspace in TCR is 4, 6, 10 and 10. In addition, optimal window sizes of PDS are all 3. We set the standard samples in range [5,30]. When the model is stable, the number of standard samples is selected for modeling based on the smallest RMSEC criteria.

**Figure 2:**
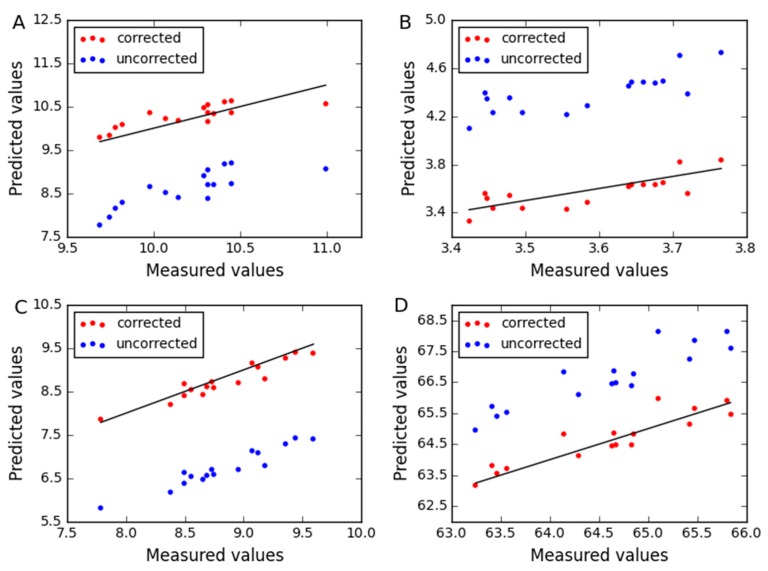
The relationship between the uncorrected and the corrected predict values for corn dataset by (**A**) moisture, (**B**) oil, (**C**) protein and (**D**) starch. The blue and red dots represent the uncorrected and the corrected predicted results for each sample, respectively.

As shown in Table 2, we can see the correlation coefficients r_pre_ and corresponding p_pre_ values, which indicate the prediction values between the master instrument and the slave instrument are linearly correlated. We can also see that the t_pre_ is greater than the t critical value. We then know the bias adjustment in predicted results should be implemented. Furthermore, the RMSE of prediction without any correction for the slave instrument shows more error of prediction than the master instrument. The corrected results of CTAI result in a significant reduction in RMSE of prediction. The same situation can be found between $y^{m}$ and ${\tilde{y}}^{n}$ in Table 2. The absolute value of t in each component is 15.437, 19.657, 19.408 and 8.762, respectively. The critical value of t is 2.131, and all results are greater than it. It is further proved that the adjustment of bias is very important. For the corn dataset, the effect of correction in CTAI is vividly described in Figure 2. It can be seen that the corrected predicted values of CTAI more close to the straight line, and RMSEP is greatly reduced.

Moreover, the results listed in Table 3 and Table 4 show the difference between the 16 predictive corn samples by different methods. In general, the results of CTAI exhibit the best performance for prediction compared to other five methods. When moisture is used as the property, CTAI achieves the lowest RMSEP (0.21095). More specifically, the RMSEP improvements provided by CTAI with respect to MSC, TCR, CCA, SBC and PDS are as high as 87.35%, 46%, 9.48%, 50.45% and 12.96%, respectively. Though there are no statistically significant differences, CTAI is greatly improved in predictive accuracy compared with CCA and TCR. There is a significant difference at the 95% confidence level between CTAI and MSC, SBC and PDS. When oil is used as the property, it can be seen that there is no significant difference between RMSEC and RMSEP in different transfer methods, so the over-fitting phenomenon does not appear. CTAI also produces the lowest RMSECV (0.08141) and RMSEC (0.08233). The results by Wilcoxon signed rank test reveal that CTAI is significantly different from MSC and TCR and has similar performance compared with CCA, SBC and PDS. It is noticeable that the RMSEP improvement rates of CTAI compared with CCA, SBC and PDS are 27.98%, 1.52% and 13.28%, respectively. Other properties are similar with the property of oil; CTAI achieves better predictive performance.

**Figure 3:**
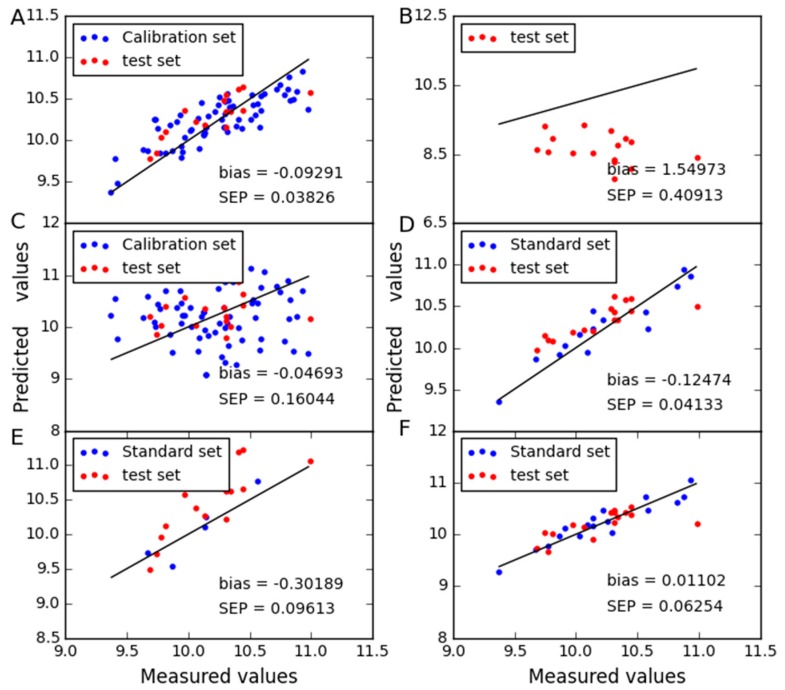
Moisture content predicted for corn dataset as determined by (**A**) CTAI, (**B**) MSC, (**C**) TCR, (**D**) CCA, (**E**) SBC and (**F**) PDS. The blue and red dots represent the results for each sample in the train set and test set, respectively.

**Figure 4:**
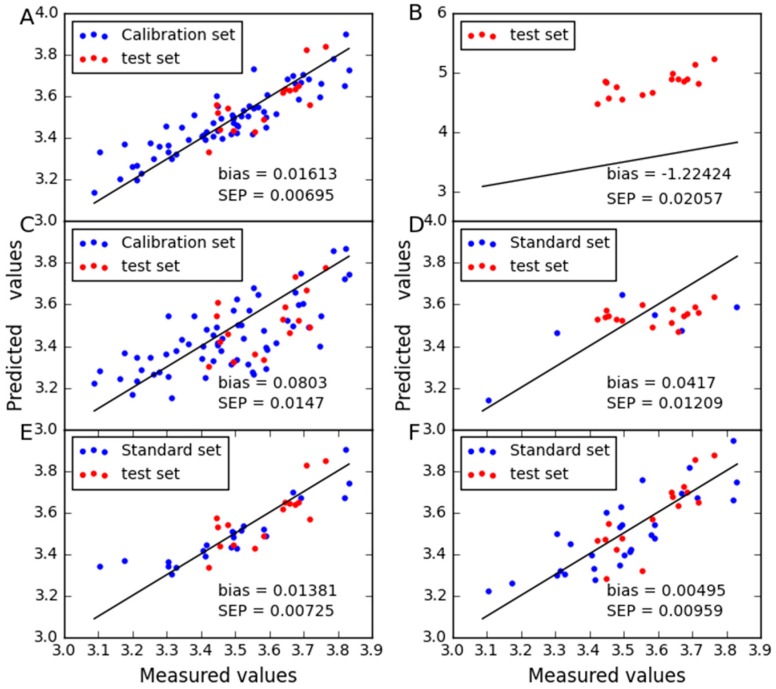
Oil content predicted for corn dataset as determined by (**A**) CTAI, (**B**) MSC, (**C**) TCR, (**D**) CCA, (**E**) SBC and (**F**) PDS. The blue and red dots represent the results for each sample in the train set and test set, respectively.

**Figure 5:**
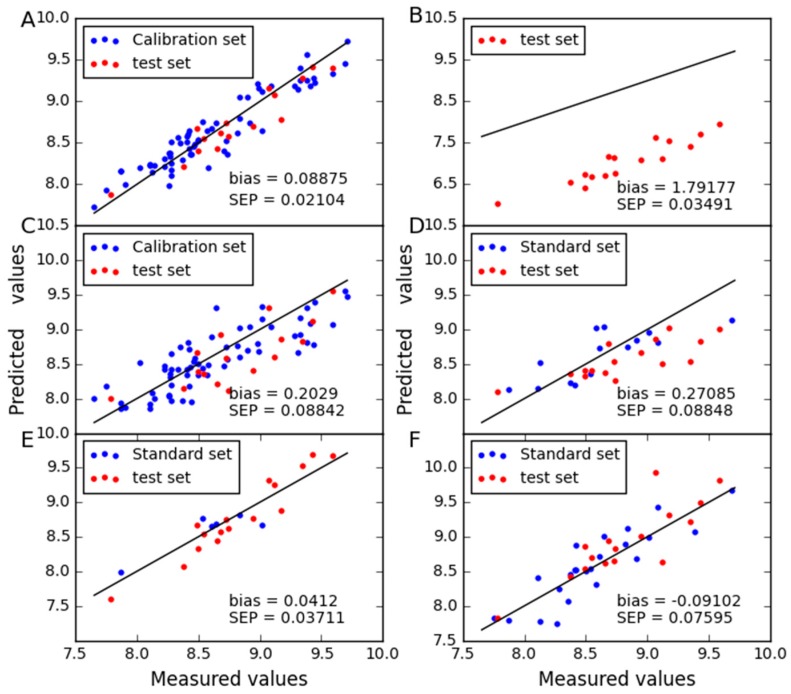
Protein content predicted for corn dataset as determined by (**A**) CTAI, (**B**) MSC, (**C**) TCR, (**D**) CCA, (**E**) SBC and (**F**) PDS. The blue and red dots represent the results for each sample in the train set and test set, respectively.

**Figure 6:**
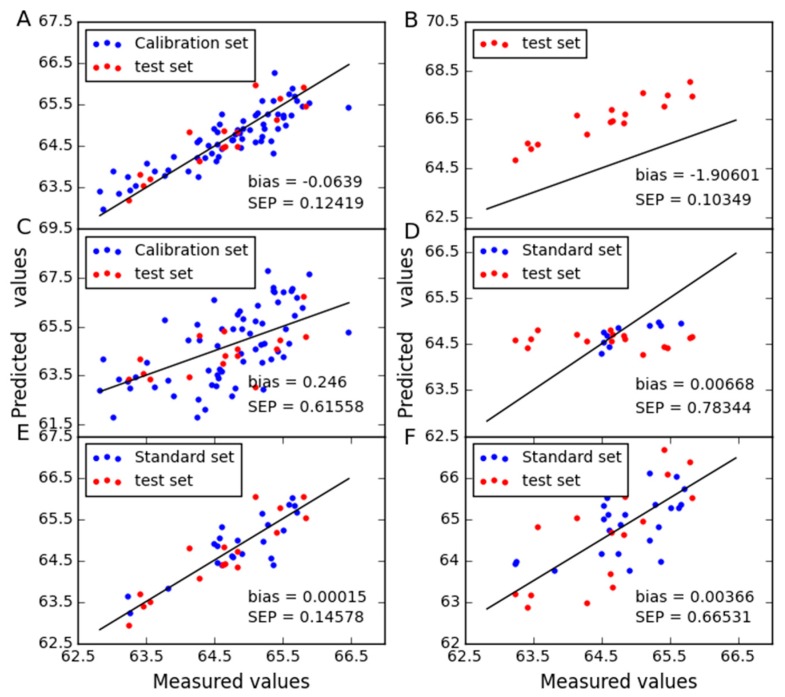
Starch content predicted for corn dataset as determined by (**A**) CTAI, (**B**) MSC, (**C**) TCR, (**D**) CCA, (**E**) SBC and (**F**) PDS. The blue and red dots represent the results for each sample in the train set and test set, respectively.

In order to compare the predictive stability of various methods, Figure 3, Figure 4, Figure 5 and Figure 6 show the plots of measured vs. predicted values for the calibration set and the test set. If the model predicts better, the point will be closer to the straight line. When moisture is used as the property, it is observed from Figure 3 that CTAI is in general closer to the straight line than the other models. It confirms that the CTAI achieves the best overall performance. When oil is used as the property, it is clear that CTAI provides satisfactory results not only in the calibration set but also in the test set. It reconfirmed that CTAI achieves more accurate prediction results. In addition, the standard error has also achieves good results in CTAI compared with others. From the discussion above, one can easily conclude that CTAI can achieve the best performance in all models and has better generalization ability.

### 2.2. Analysis of the Wheat Dataset

The RMSEP of the PLS model is listed in Table 1. We can see that the predicted performance of the instrument B1 is better than B3 and the instrument B3 is better than B2. Thus, three combinations (B1-B2; B1-B3; B3-B2) of the instruments B1, B2 and B3 are used to analyze the wheat dataset. The first instrument of every combination stands for master instrument and the second instrument stands for slave instrument. For PLS model, the optimal number of latent variables is 14, 15 and 15, respectively, and the corresponding optimal dimensionality of the subspace in TCR is 17, 12 and 17, respectively. Moreover, the optimal number of window sizes for B1-B2, B1-B3 and B3-B2 is 3, 9 and 13, respectively.

**Figure 7:**
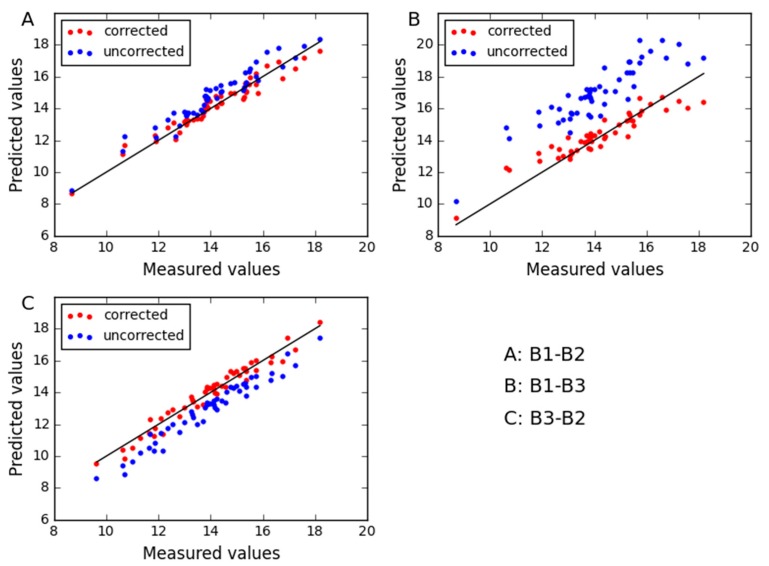
The relationship between the uncorrected and the corrected predict values for wheat dataset by (**A**) B1-B2, (**B**) B1-B3 and (**C**) B3-B2. The blue and red dots represent the uncorrected and the corrected predicted results for each sample, respectively.

For the three combinations of instruments (B1-B2; B1-B3; B3-B2), we can see between $y^{m}$ and ${\tilde{y}}^{n}$ the correlation coefficients r_pre_ are large and p_pre_ are close to zero in Table 2. Hence, there is a linear relationship between the predicted values of the two instruments for wheat dataset. For all combinations, the absolute value of t is greater than t_critical_value_. So there is a significant bias between uncorrected predicted values of the slave instrument and predicted values of the master instrument. So we can correct the predicted values of the slave instrument by affine transformation. The experimental results show that the prediction performance of CTAI is significantly enhanced. We found the same phenomenon for the uncorrected prediction values of the slave instrument relative to the master instrument actual values. Furthermore, for the predicted performance of CTAI, Figure 7 shows the difference between uncorrected and corrected predicted values for B1-B2, B1-B3 and B3-B2. It can be seen that CTAI plays an important role in the correction of predicted values.

In addition, Table 3 lists the results of different methods for calibration set and test set. For the B1-B2, CTAI produces the lowest RMSEP (0.41419) and the second lowest RMSEC (0.55682). For PDS and CCA, it is worth noting that RMSEP is significantly larger than RMSEC. Therefore, the predictive performance of PDS and CCA are poor under this setting. Further, a statistical testing is utilized to evaluate the RMSEP difference between the CTAI and other methods for the wheat dataset. The Wilcoxon signed rank sum test was performed and at the significance level alpha = 0.05. It can be seen from Table 4 that there is a statistically significant difference compared with CCA, SBC and PDS. In addition, the improvement rates of prediction provided by CTAI for MSC and TCR are up to 55.07% and 52.32%, respectively. For the combination (B1-B3), CTAI displays the lowest RMSEP (0.68215), followed by TCR (0.72996) and SBC (0.79294). For PDS, we can see that under-fitting still existed under this setting, and for CCA, this phenomenon also exists, but it is not particularly serious. The results by Wilcoxon signed rank test show that CTAI is significantly different from MSC, CCA, SBC and PDS (shown in Table 4). Compared with TCR, RMSEP improvement rates of CTAI can reach 6.55%. For the last combination, both RMSEP and RMSEC achieve the best predicted results. Further, except for PDS, the differences between CTAI and other models are statistically significant at the 95% confidence level. Compared with PDS, the RMSEP improvements of CTAI are as high as 79.05%. It is also worth noting that there is no under-fitting phenomenon in PDS under the current setting, but the predicted results are still poor. Therefore, the predictive performance of PDS is worse for wheat datasets under the current model.

**Figure 8:**
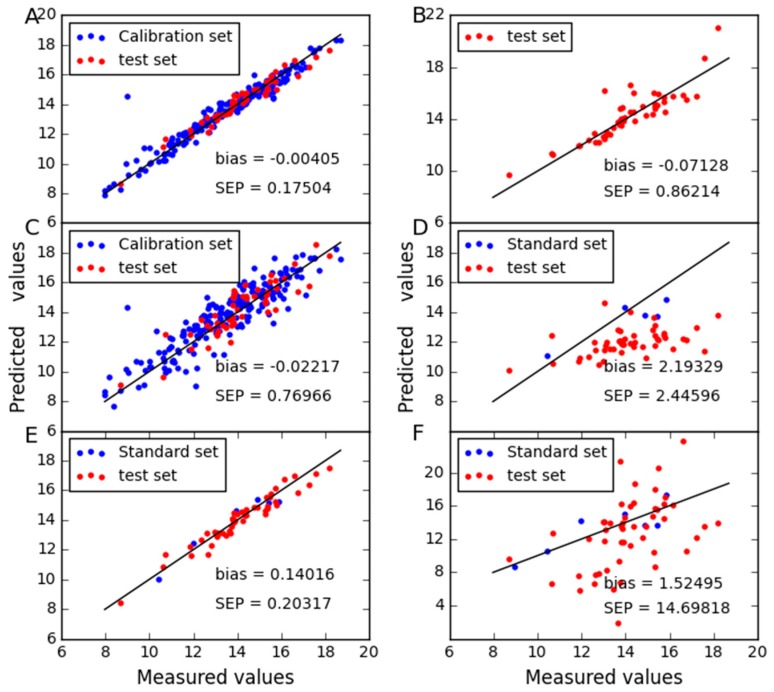
Protein content predicted between instruments B1 and B2 for wheat dataset as determined by (**A**) CTAI, (**B**) MSC, (**C**) TCR, (**D**) CCA, (**E**) SBC and (**F**) PDS. The blue and red dots represent the results for each sample in the train set and test set, respectively.

**Figure 9:**
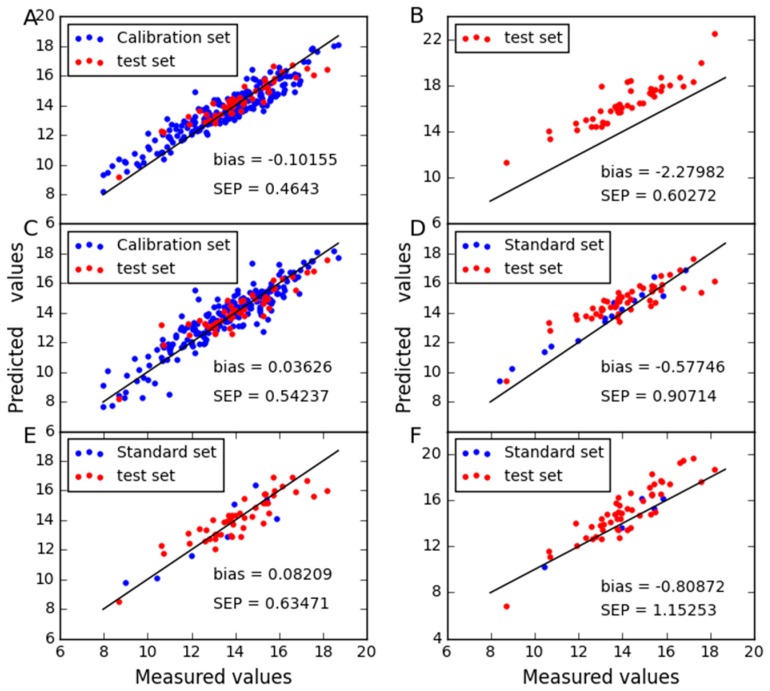
Protein content predicted between instruments B1 and B3 for wheat dataset as determined by (**A**) CTAI, (**B**) MSC, (**C**) TCR, (**D**) CCA, (**E**) SBC and (**F**) PDS. The blue and red dots represent the results for each sample in the train set and test set, respectively.

**Figure 10:**
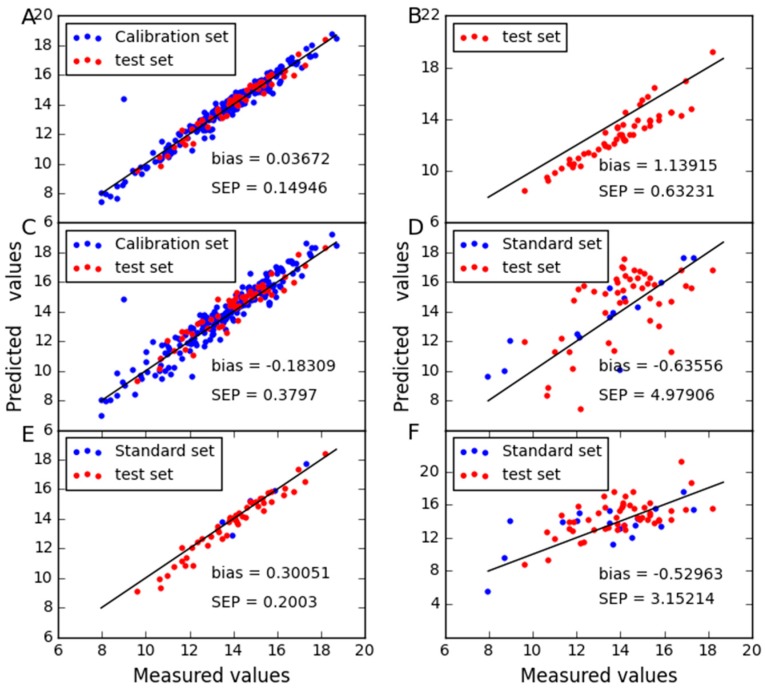
Protein content predicted between instruments B3 and B2 for wheat dataset as determined by (**A**) CTAI, (**B**) MSC, (**C**) TCR, (**D**) CCA, (**E**) SBC and (**F**) PDS. The blue and red dots represent the results for each sample in the train set and test set, respectively.

To further display the predictive abilities of different models, the correlation between measured and predicted values obtained in Figure 8, Figure 9 and Figure 10. Zero differences between measured and predicted values result in points over the straight line of the plot. It can be seen that good correlations are found between expected and predicted concentrations, which confirm the good performance of CTAI. CTAI achieved the lowest standard error for three combinations. Moreover, the predictive abilities of PDS and CCA are poor for wheat dataset. For SBC, PDS and CCA, they require standard samples and TCR requires reference values of the slave instrument samples, both of which are expensive and difficult to obtain. Obviously, this means that CTAI shows much more outstanding performance.

## 3. Materials and Methods

### 3.1. Dataset Description

#### 3.1.1. Corn Dataset

**Figure 11:**
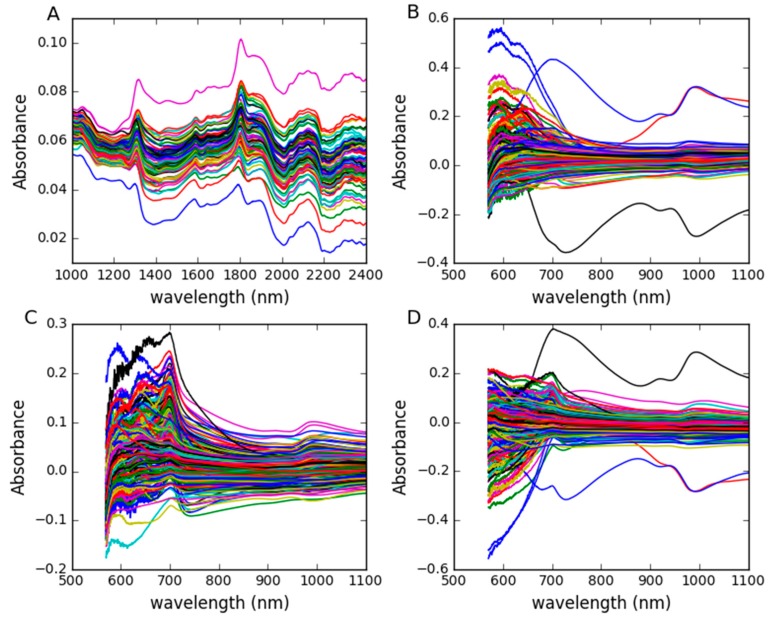
(**A**) Spectral differences between m5 and mp6 of corn samples; (**B**) spectral differences between B1 and B2 of wheat samples; (**C**) spectral differences between B1 and B3 of wheat samples; (**D**) spectral differences between B2 and B3 of wheat samples.

The corn dataset, which contains 80 samples, was measured on three NIR spectrometers (m5, mp5 and mp6). Each sample consists of four components: Moisture, oil, protein, and starch. The wavelength range is 1100–2400 nm with interval 2 nm (700 channels). The spectra measured in m5spec were used as the master spectra, and the spectra measured by mp6spec were used as the secondary spectra. The data can be obtained from http://www.eigenvector.com/data/Corn/. The dataset was divided into a calibration set of 64 samples and a test set of 16 samples based on Kennard-Stone (KS) algorithm. The NIR spectra are shown in Figure 11A, which represents the difference between m5 and mp6.

#### 3.1.2. Wheat Dataset

The wheat dataset was used as the shootout data for the International Diffuse Conference 2016, and the protein content was chosen as the property. Related information about the wheat dataset at http://www.idrc-chambersburg.org/content.aspx?page_id=22&club_id=409746&module_id=191116 can be easily accessed. 248 samples of the wheat dataset from three different NIR instrument manufacturers (B1, B2 and B3) were analyzed. According to KS algorithm, 198 samples were chosen as the calibration set and the remainder of samples formed the test set. The wavelength range is 570–1100 nm with an interval of 0.5 nm. The spectral difference between B1 and B2 is shown in Figure 11B. The spectral difference between B1 and B3 is shown in Figure 11C. The spectral difference between B2 and B3 is shown in Figure 11D.

### 3.2. Determination of the Optimal Parameters

Latent variables of PLS in CTAI are allowed to take values in the set [1,15], and it is determined by the 10-fold cross-validation. The optimal number of latent variables is selected only when the lowest RMSECV.

Five methods were used for comparison, where the latent variable range and parameter optimization all of SBC, CCA, PDS and MSC in PLS are consistent with CTAI. In particular, the window size in PDS is searched for from 3 to 16 in increments of 2, and is selected by 5-fold cross-validation. In addition, the dimensionality of the TCA space in TCR is estimated in the range [1,24] and the optimization criteria are consistent as described in [24].

### 3.3. Model Performance Evaluation

In this experiment, root mean squared error RMSE is employed as indicators for parameter selection and model evaluation. Furthermore, RMSEC is the training error, RMSECV denotes the cross-validation error and RMSEP indicates the prediction error of the test set. The RMSE calculation method is written as:

(1) $$\mathrm{RMSE}=\sqrt{{\left(y-\hat{y}\right)}^{T}\left(y-\hat{y}\right)/n}$$

where $\hat{y}$ is the predict value, y is the measured value and *n* represents the number of samples.

Bias and standard error (SE) are also utilized as reference indicators for model evaluation. The bias and SE are as follows:

(2) $$\{\begin{array}{c} \mathrm{bias}=\sum_{i}^{n}\left(y_{i}-{\hat{y}}_{i}\right)/n\\ \mathrm{SE}\;=\;\sqrt{\left({\left(y-\hat{y}\right)}^{T}\left(y-\hat{y}\right)-\mathrm{bias}\right)/n} \end{array}$$

Moreover, the Pearson correlation coefficient and corresponding test is used to determine if there is a linear relationship between the master instrument and the slave instrument. One-Sample *t*-Test is also utilized to determine whether a bias adjustment in predicted results should be implemented [11].

In order to compare CTAI and other methods further, another important parameter (*h*) is cited in order to compare the rate of improvement, defined as follows:

(3) $$h=\left(1-\frac{\mathrm{RMSEP}}{{\mathrm{RMSEP}}_{\mathrm{other}}}\right)\times 100\%$$

where RMSEP represents the prediction error of CTAI and ${\mathrm{RMSEP}}_{\mathrm{other}}$ represents the others.

In addition, the Wilcoxon signed rank sum test at the 95% confidence level is used to determine whether there is a significant difference between CTAI and the others.

### 3.4. Computational Environment

All experimental procedures were implemented on a personal computer by python language, software version python 2.7, and run on an acer notebook with a 2.60 GHz Intel (R) Core (TM) i5-3230M CPU, 8 GB RAM and a Microsoft Windows 7 operating system (Acer Incorporated, Taiwan, China). Normalization and cross-validation are performed using the sklearn package. The Wilcoxon signed rank test is implemented using the scipy package and other programs are implemented by the individual.

### 3.5. Calibration Transfer

#### 3.5.1. Notation

In the following text, matrices are represented by bold capital letters (e.g., **X**), column vectors by bold lower case letters (e.g., **y**) and scalars by italic letters (e.g., *a*). The transposition operation is indicated by superscript ^T^.

#### 3.5.2. Overview of PLS

PLS is used to establish the linear relationship between the input space and the response space. The purpose of the PLS model is to ensure the optimal number of latent variables. The latent variables are linear combinations of the primitive variables. The latent variables are calculated in this way so that they contain a maximum of relevant information concerning the relation between X and y. Mathematically, this is shown by the following objective function.

(4) $$\begin{array}{l} H=\underset{w}{\mathrm{argmax}}\;\;\;\;\;\mathrm{cov}\langle Xw,\;y\rangle \\ \mathrm{subject}\;\mathrm{to}\;\;\;{\left||w|\right|}_{2}=1 \end{array}$$

where w represents the weight vector. This objective is a maximization problem under one constraint, which can be settled in virtue of the Lagrange multiplier method.

Assuming a PLS model is built between spectral matrix $X\in {ℜ}^{n\times p}$ and concentration vector $y\in {ℜ}^{n\times 1}$, the model is named PLS1 (*n* denotes the number of samples and *p* represents the optimal numbers of latent variables). In the algorithm, the first weighting vector must be the primary eigenvector of the matrix $X^{T}yy^{T}X$. From the second latent variable on, it requires the following latent variables to be orthogonal (uncorrelated) to the former ones. Hence, the following weighting vectors will be the dominant eigenvectors of the matrix $X^{T}yy^{T}X$; also, repeat a sequence of the steps until convergence. The PLS1 is built using the following model:

(5) $$\left\{ \begin{array}{l} X^{n\times p}=T^{n\times A}{\left(P^{p\times A}\right)}^{T}+E^{n\times p}\\ y^{n\times 1}=T^{n\times A}{\left(Q^{1\times A}\right)}^{T}+F^{n\times 1} \end{array}\right.$$

where **T** is the score matrix and **P** and **Q** represent the **X**-loading matrix and **y**-loading vector, respectively; **E** and **F** denote the matrix of residuals; A is the optimal number of principal components over the master instrument PLS model.

Finally, the regression coefficient β of the model can be written as follows:

(6) $$\beta =W{\left(P^{T}W\right)}^{-1}Q^{T}$$

where $W=[w_{1},w_{2},\ldots ,w_{A}]$ represents the weight matrix.

#### 3.5.3. Affine Transformation

**Figure 12:**
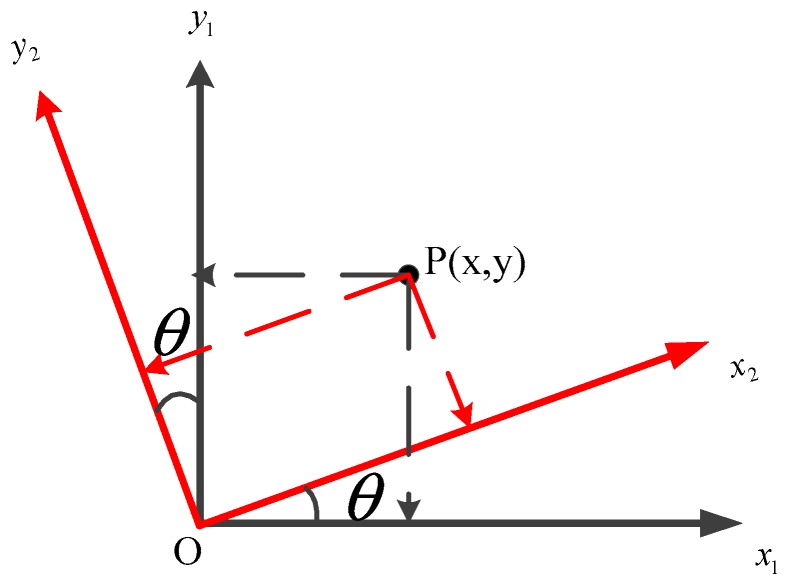
Derivation of affine transformation. In the coordinate system, the counterclockwise rotation of P is equivalent to the clockwise rotation of the coordinate system.

This paper focuses on the rotation and translation properties of two-dimensional affine transformation [39]. After transformation, the original line is still a straight line and the original parallel line is still parallel. Affine transformation is a transformation of coordinates. Based on Figure 12, the derivation is written as follows:

Point P in the original coordinate system (black) is (x, y). A counterclockwise rotation of the point P is equivalent to clockwise rotation of the coordinate system. Thus, the point P in the black coordinate system is equivalent with the point P in the red coordinate system after the rotation. Based on this conclusion, we can determine the coordinates of the point P by simple stereo geometry, and then add the offset of the X-axis and the Y-axis based on this position; the formula is as follows:

(7) $$\left\{ \begin{array}{l} x^{\prime }=x\cos\theta -y\sin\theta +\Delta x\\ y^{\prime }=y\cos\theta +x\sin\theta +\Delta y \end{array}\right.$$

where θ is the angle of rotation, Δx is the offset on the X axis and Δy is the offset on the Y axis; $x^{\prime }$ and $y^{\prime }$ are coordinate in the new coordinate system.

#### 3.5.4. Calibration Transfer Method based on Affine Transformation

Based on the inputs and outputs $\left\{X^{m},y^{m}\right\}$ from the master instrument, and the inputs $\left\{X^{s}\right\}$ from the slave instrument, our task is to predict the unknown outputs $\left\{{\hat{y}}^{s}\right\}$ in the slave instrument. We assume that $X^{m}$ and $X^{s}$ are the spectra of two similar substances, and $y^{m}$ and ${\hat{y}}^{s}$ are in the same range. Due to the difference between two instruments, the observed spectral data are different. The observations from the perspective of the master instrument model are as follows:

(8) $$\left\{ \begin{array}{l} {\hat{y}}^{m}=F(X^{m},\beta^{m})=\sum_{i=1}^{A}t_{i}^{m}q_{i}^{m}\\ {\tilde{y}}^{s}=F(X^{s},\beta^{m})=\sum_{i=1}^{A}{\tilde{t}}_{i}^{s}q_{i}^{m} \end{array}\right.$$

where F is the linear prediction function, which is obtained by partial least squares in this paper; $\beta^{m}$ is the coefficient of the master model and ${\hat{y}}^{m}$, $t_{i}^{m}$ and $q_{i}^{m}$ are the predicted values, the *i*-th column score vector and the loading vector, respectively. Accordingly, ${\tilde{y}}^{s}$ and ${\tilde{t}}_{i}^{s}$ are the biased predicted values and the *i*-th biased column score vector for the slave instrument, respectively.

Therefore, the score vectors and predicted values both of the two instruments are different. As a result, there is a certain bias that needs to be corrected in the coefficient between the score vector and predicted values.

When correcting the bias, direct calculation will produce large errors. In order to solve this problem, we need to transform the score vectors and predicted values of the master and slave instrument into the range [0, 1] and thus keep the same scale between different values. The corresponding equations are given as follows:

(9) $$\{\begin{array}{l} t_{}^{m-\mathrm{norm}}=(t_{i}^{m}-\min(t_{i}^{m}))/(\max(t_{i}^{m})-\min(t_{i}^{m}))\\ {\hat{y}}^{m-\mathrm{norm}}=({\hat{y}}_{}^{m}-\min({\hat{y}}_{}^{m}))/(\max({\hat{y}}_{}^{m})-\min({\hat{y}}_{}^{m}))\\ {\tilde{t}}_{}^{s-\mathrm{norm}}=({\tilde{t}}_{}^{s}-\min({\tilde{t}}_{i}^{s}))/(\max({\tilde{t}}_{i}^{s})-\min({\tilde{t}}_{i}^{s}))\\ {\tilde{y}}^{s-\mathrm{norm}}=({\tilde{y}}^{s}-\min({\tilde{y}}^{s}))/(\max({\tilde{y}}^{s})-\min({\tilde{y}}^{s})) \end{array}$$

where $t_{i}^{m-\mathrm{norm}}$ and ${\hat{y}}^{m-\mathrm{norm}}$ are the normalized score vector and the predicted values of the master instrument, respectively; ${\tilde{t}}_{i}^{s-\mathrm{norm}}$ and ${\tilde{y}}^{s-\mathrm{norm}}$ are the normalized and biased score vector and predicted values, respectively.

Two linear regression equations between score vector and predicted values are as follows:

(10) $$\left\{ \begin{array}{l} {\hat{y}}^{m-\mathrm{norm}}=t_{i}^{m-\mathrm{norm}}\tan\theta_{i}^{m}+b_{i}^{m}\\ {\tilde{y}}^{s-\mathrm{norm}}={\tilde{t}}_{i}^{s-\mathrm{norm}}\tan{\tilde{\theta }}_{i}^{s}+{\tilde{b}}_{i}^{s} \end{array}\right.$$

where $\tan\theta_{i}^{m}$ and $\tan{\tilde{\theta }}_{i}^{s}$ are the regression coefficients (slopes) computed on the two instrument; $b_{i}^{m}$ and ${\tilde{b}}_{i}^{s}$ are the intercepts.

**Figure 13:**
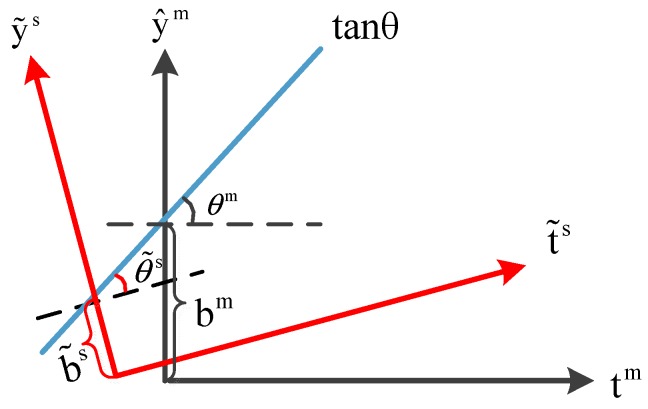
Theory of CTAI. tanθ is the coefficient between the feature vector and the predicted values. The angles and deviations observed under different instruments are different. We correct the predicted value of the slave instrument with the rotation and translation of affine transformation.

In order to more intuitively reflect the difference between two instruments, it can be better understood from Figure 13. The blue line is the regression coefficient between the score vector and predicted values. The black and red coordinate systems are the observations of the master and slave instrument, and there is a difference from different observations.

The unknown angles and biases between two instruments are solved as follows:

Firstly, the regression coefficient $\beta^{m}$, the weight $W^{m}$ and loading $P^{m}$ matrix of PLS are obtained.

Secondly, a linear regression both of master and slave instrument is performed and slopes and intercepts are determined, respectively.

On the grounds of the PLS model, the score matrices and predicted values are calculated as shown below:

(11) $$\left\{ \begin{array}{ll} T^{m} & =X^{m}W^{m}{\left(P^{m}W^{m}\right)}^{-1},\;\;\;{\hat{y}}^{m}=X^{m}\beta^{m}\\ {\tilde{T}}^{s} & =X^{s}W^{m}{\left(P^{m}W^{m}\right)}^{-1}\;\;,\;\;\;\;{\tilde{y}}^{s}=X^{s}\beta^{m} \end{array}\right.$$

where $T^{m}$ and ${\tilde{T}}^{s}$ represent the score matrices of two instruments.

The score matrix $T^{m}$, predicted values ${\hat{y}}^{m}$, the score matrix ${\tilde{T}}^{s}$ and predicted values ${\tilde{y}}^{s}$ are pre-processed using Equation (9).

According to score vector of each column and predicted values, the least square is used to compute the corresponding slopes and intercepts, respectively. The equations are as follows:

(12) $${\left\{ \begin{array}{l} \underset{\theta_{i}^{m},b_{i}^{m}}{\min}\left\|{\hat{y}}^{m-\mathrm{norm}}-T_{\mathrm{aug}}^{m}*\left[\begin{array}{c} \tan\theta_{i}^{m}\\ b_{i}^{m} \end{array}\right]\right\|\\ \underset{{\tilde{\theta }}_{i}^{s},{\tilde{b}}_{i}^{s}}{\;\;\;\min}{\left\|{\tilde{y}}^{s-\mathrm{norm}}-T_{\mathrm{aug}}^{s}*\left[\begin{array}{c} \tan{\tilde{\theta }}_{i}^{s}\\ {\tilde{b}}_{i}^{s} \end{array}\right]\right\|}^{2} \end{array}\right.}^{2}$$

where $T_{\mathrm{aug}}^{m}$ is an augmented matrix $\left[\begin{array}{cc} t_{i}^{m-\mathrm{norm}}, & 1 \end{array}\right]$; $T_{\mathrm{aug}}^{s}$ is an augmented matrix $\left[\begin{array}{cc} {\tilde{t}}_{i}^{s-\mathrm{norm}}, & 1 \end{array}\right]$; **1** is the column vector with all ones.

Finally, the angle and biases between the two instruments are obtained. The equations for calculating the angles and biases are as follows:

(13) $$\left\{ \begin{array}{l} \Delta \theta_{i}=\theta_{i}^{m}-{\tilde{\theta }}_{i}^{s}\\ \Delta b_{i}=b_{i}^{m}-{\tilde{b}}_{i}^{s} \end{array}\right.$$

where $\Delta \theta_{i}$ is the angle of the two coefficients; $\Delta b_{i}^{}$ is the corresponding bias.

The score matrix and predicted values of the test set are extracted by Equation (11).

The angles and biases obtained by Equation (13) are brought into the affine transformation to correct the predicted values. Since the rotation angle is relative to the origin of the coordinate, each sample needs to be adjusted before rotation. The equation is shown as follows:

(14) $${\hat{U}}_{i}={\tilde{U}}_{i}M_{i}$$

where the matrix $M_{i}=\left[\begin{array}{ccc} \lambda_{t}\cos\Delta \theta_{i} & \lambda_{t}\sin\Delta \theta_{i} & 0\\ -\lambda_{y}\sin\Delta \theta_{i} & \lambda_{y}\cos\Delta \theta_{i} & 0\\ 0 & b_{i}^{m} & 1 \end{array}\right]$,${\tilde{U}}_{i}=\left[{\tilde{t}}_{i}^{s-\mathrm{test}},{\tilde{y}}^{s-\mathrm{test}},1\right]$ and ${\hat{U}}_{i}=\left[{\hat{t}}_{i}^{s-\mathrm{test}},{\hat{y}}^{s-\mathrm{test}},1\right]$. In addition, $\lambda_{t}=\left[\left({\tilde{t}}_{i}^{s-\mathrm{test}}-\min({\tilde{t}}_{i}^{s})\right)/(\max({\tilde{t}}_{i}^{s})-\min({\tilde{t}}_{i}^{s}))+\min({\tilde{t}}_{i}^{s})\right]\times (\max({\tilde{t}}_{i}^{s})-\min({\tilde{t}}_{i}^{s}))$ and $\lambda_{y}=\left[({\tilde{y}}_{i}^{s-\mathrm{test}}-\min({\tilde{y}}^{s}))/(\max({\tilde{y}}^{s})-\min({\tilde{y}}^{s}))+\min({\tilde{y}}^{s})\right]\times (\max({\tilde{y}}^{s})-\min({\tilde{y}}^{s}))$ represent the corresponding scaling factors for feature vector and predicted values, respectively; ${\tilde{t}}_{i}^{s-\mathrm{test}}$ and ${\tilde{y}}_{}^{s-\mathrm{test}}$ are biased score vector and predicted values of the test set, respectively; ${\hat{y}}^{s-\mathrm{test}}$ is corrected predicted values; ${\hat{t}}_{i}^{s-\mathrm{test}}$ is corrected score vector.

Each column score vector and predicted values are solved separately, and a prediction matrix is obtained. The mean of the prediction matrix is the final predicted values.

Therefore, according to the expansion of the predicted values, $\beta^{s}$ is as follows:

(15) $$\beta^{s}={\left({\left(X^{s}\right)}^{T}X^{s}\right)}^{-1}{\left(X^{s}\right)}^{T}(\sum_{i}^{A}({\tilde{t}}_{i}^{s-\mathrm{test}}*\lambda_{t}*\sin\Delta \theta_{i}+({\tilde{y}}_{}^{s-\mathrm{test}}-{\tilde{b}}_{i}^{s}*1)*\lambda_{y}*\cos\Delta \theta_{i}+b_{i}^{m})/A)$$

#### 3.5.5. Summary of CTAI

Given calibration set of the master $\left(X_{\mathrm{cal}}^{m},y_{\mathrm{cal}}^{m}\right)$, calibration set of the slave $X_{\mathrm{cal}}^{s}$ and test set $\left(X_{\mathrm{test}}^{s},y_{\mathrm{test}}^{s}\right)$.The PLS model is built on the calibration set $\left(X_{\mathrm{cal}}^{m},y_{\mathrm{cal}}^{m}\right)$ and the coefficient $\beta^{m}$; the weight matrix $W^{m}$ and the loading matrix $P^{m}$ can be obtained.Modeling of affine transformation; it consists of the two datasets $\left(X_{\mathrm{cal}}^{m},y_{\mathrm{cal}}^{m}\right)$ and $X_{\mathrm{cal}}^{s}$. (a)Computing $\left(T_{\mathrm{cal}}^{m},{\hat{y}}_{\mathrm{cal}}^{m}\right)$ and $\left({\tilde{T}}_{\mathrm{cal}}^{s},{\tilde{y}}_{\mathrm{cal}}^{s}\right)$ of master and slave instrument by Equation (11).(b)$\left(T_{\mathrm{cal}}^{m},{\hat{y}}_{\mathrm{cal}}^{m}\right)$ and $\left({\tilde{T}}_{\mathrm{cal}}^{s},{\tilde{y}}_{\mathrm{cal}}^{s}\right)$ are normalized separately by Equation (9).(c)$(\tan\theta_{i}^{m},b_{i}^{m})$ and $\left(\tan{\tilde{\theta }}_{i}^{s},{\tilde{b}}_{i}^{s}\right)$ are calculated by Equation (12).(d)Computing $\Delta \theta_{i}$ angle and $\Delta b_{i}$ bias between master and slave instrument by Equation (13).Prediction.(a)$\left({\tilde{T}}_{\mathrm{test}}^{s},{\tilde{y}}_{\mathrm{test}}^{s}\right)$ is obtained by Equation (11).(a)The matrix $M_{i}$ is introduced to correct predicted values by Equation (14).(c)The corrected prediction values are accumulated. The mean values are the last result.

## 4. Conclusions

In this study, the relationship of regression coefficients between feature vector and predicted values on different instruments was investigated and CTAI was proposed for calibration transfer based on affine invariance without transfer standards (CTAI). Based on the PLS model of the master instrument, the score matrix and the predicted values of the master spectra, the pseudo score matrix and the pseudo predicted values of the slave spectra are obtained. Then, angles and biases between the coefficients of the master instrument and the corresponding coefficients of the slave instrument are computed. Finally, new samples are corrected by affine transformation. Different transfer methods are tested with two NIR datasets, CTAI achieves the lowest RMSEP and standard error, and the results of statistical difference indicate that CTAI is generally better than other methods, which proves that CTAI is successfully used to correct the difference on different instruments. Hence, the proposed method may provide an efficient way for calibration transfer when standard samples are unavailable in practical applications.

## Figures and Tables

**Table 1:** Summary of the partial least squares (PLS) models and properties.

Instrument	Reference Values	RMSEC^m^	RMSEP^m^	RMSECV_min_ (LV)	Bias^m^	r^m^	p^m^
m5spec	moisture	0.00599	0.00764	0.01066(14)	0.0008	0.99973	2.6 × 10^−24^
m5spec	oil	0.02686	0.05664	0.05049(15)	−0.01327	0.9332	1.3 × 10^−7^
m5spec	protein	0.0507	0.10066	0.11012(15)	0.02814	0.97632	1 × 10^−10^
m5spec	starch	0.09539	0.18993	0.19227(15)	0.01789	0.97464	1.6 × 10^−10^
mp6spec	moisture	0.09991	0.15637	0.14775(10)	−0.02678	0.92083	4.2 × 10^−7^
mp6spec	oil	0.06052	0.09098	0.09872(12)	0.01868	0.87697	8.2 × 10^−6^
mp6spec	protein	0.10101	0.13338	0.15043(12)	0.02128	0.96659	1.1 × 10^−9^
mp6spec	starch	0.27636	0.26723	0.35978(9)	0.02124	0.93136	1.6 × 10^−7^
B1	protein	0.3288	0.33254	0.50337(15)	0.00906	0.98508	2.3 × 10^−38^
B2	protein	0.21636	0.83755	0.32441(15)	−0.13124	0.8485	7.2 × 10^−15^
B3	protein	0.30288	0.51567	0.43896(15)	−0.034	0.96009	3.2 × 10^−28^

RMSEC^m^: Root Mean Square Error of calibration set; RMSEP^m^: Root Mean Square Error of test set; RMSECV_min_: Minimum Root Mean Square Error of Cross-Validation; LV: The optimal number of latent variables is selected only with the lowest RMSECV; r^m^: Pearson correlation coefficient for predicted vs. actual values; p^m^: p values corresponding to the Pearson correlation coefficient is obtained by test.

**Table 2:** Summary of the relevant results between uncorrected and CTAI corrected.

Instrument Reference Values		m5spec*-mp6spec				B1*-B2	B1*-B3	B3*-B2
		Moisture	Oil	Protein	Starch	Protein		
${\hat{y}}^{m}\mathrm{vs}\;{\tilde{y}}^{s}$	RMSEP^u^_pre_	1.60705	0.7989	2.06797	2.11743	0.69894	2.92541	1.23368
	RMSEP_pre_	0.21255	0.06922	0.13195	0.33358	0.31537	0.62632	0.65398
	k_pre_	0.6498	0.77129	0.94553	0.82527	0.88809	0.76290	0.86909
	r_pre_	0.81644	0.89598	0.96286	0.92197	0.97594	0.87695	0.93715
	p_pre_	1.1 × 10^−4^	2.6 × 10^−6^	2.3 × 10^−9^	3.8 × 10^−7^	2 × 10^−33^	6.8 × 10^−17^	1.3 × 10^−23^
	t_pre_	−15.429	19.335	−19.147	8.838	2.292	10.684	-3.826
$y^{m}\mathrm{vs}\;{\tilde{y}}^{s}$	RMSEP^u^	1.60762	0.81532	2.09665	2.10291	0.71977	2.90011	1.08008
	RMSEP	0.21095	0.08233	0.16614	0.34714	0.41419	0.68215	0.38446
	k	0.65191	0.53297	0.98736	0.79329	0.96898	0.85693	0.93896
	r	0.81922	0.78858	0.95844	0.91487	0.96770	0.89517	0.97796
	p	1.0 × 10^−4^	2.8 × 10^−4^	5.1 × 10^−9^	6.9 × 10^−7^	2.2 × 10^−30^	1.8 × 10^−18^	2.5 × 10^−34^
	t	−15.437	19.657	−19.408	8.762	2.256	10.649	−3.701
t_critical_value_		2.131	2.131	2.131	2.131	2.01	2.01	2.01

*: The master instrument; RMSEP^u^_pre_: RMSEP of uncorrected slave instrument relative to primary instrument prediction; RMSEP_pre_: RMSEP of CTAI corrected slave instrument relative to primary instrument prediction; k_pre_: The slope between predicted values of uncorrected slave instrument and primary prediction; r_pre_: Correlation coefficient of uncorrected slave prediction relative to master prediction; p_pre_: p values corresponding to the Pearson correlation coefficient are obtained by test; t_pre_: The result of One-Sample *t*-Test between uncorrected slave prediction and master prediction; RMSEP^u^: RMSEP of uncorrected slave instrument relative to primary actual values; RMSEP: RMSEP of CTAI corrected slave instrument relative to primary actual values; k: The slope between predicted values of uncorrected slave instrument and primary actual values; r: Pearson correlation coefficient of uncorrected slave prediction relative to primary actual values; p: p values corresponding to the Pearson correlation coefficient are obtained by test; t: The result of One-Sample *t*-Test between uncorrected slave prediction and master actual values; t_critical_value_: The t critical value for *n*–1 degrees of freedom at the significance level alpha = 0.05.

**Table 3:** Summary of Root Mean Square Error of test set (RMSEP) and Root Mean Square Error of calibration set (RMSEC) of different methods. The m5spec was used as the master spectra, and the mp6spec was used as the secondary spectra for corn dataset. The protein content was chosen as the property for wheat dataset.

Method		CTAI	MSC	TCR	CCA	SBC	PDS
moisture	RMSEC	0.22646	1.92839	0.61873	0.15996(14^a^)	0.18506(5^a^)	0.14742(17^a^)
	RMSEP	0.21095	1.6689	0.39066	0.23304(14^a^)	0.42574(5^a^)	0.24238(17^a^)
oil	RMSEC	0.08141	1.21647	0.14543	0.15764(6^a^)	0.08423(23^a^)	0.10794(28^a^)
	RMSEP	0.08233	1.23209	0.14225	0.11432(6^a^)	0.08361(23^a^)	0.09495(28^a^)
protein	RMSEC	0.17247	1.77294	0.28297	0.27860(14^a^)	0.17422(6^a^)	0.24662(23^a^)
	RMSEP	0.16614	1.80087	0.35223	0.39535(14^a^)	0.19101(6^a^)	0.28193(23^a^)
starch	RMSEC	0.39517	1.89165	1.21093	0.33937(10^a^)	0.38426(23^a^)	0.62099(23^a^)
	RMSEP	0.34714	1.93129	0.79852	0.85704(10^a^)	0.36969(23^a^)	0.78977(23^a^)
B1*-B2	RMSEC	0.55682	1.31153	0.99246	1.11889(5^a^)	0.48509(6^a^)	1.3676(7^a^)
	RMSEP	0.41419	0.92194	0.86881	2.68469(5^a^)	0.4677(6^a^)	4.09019(7^a^)
B1*-B3	RMSEC	0.81895	2.91695	0.84682	0.68529(15^a^)	1.00007(8^a^)	0.57858(5^a^)
	RMSEP	0.68215	2.40587	0.72996	1.10564(15^a^)	0.79294(8^a^)	1.33547(5^a^)
B3*-B2	RMSEC	0.54753	1.25096	0.76972	1.57073(14^a^)	0.56236(5^a^)	2.1039(8^a^)
	RMSEP	0.38446	1.38468	0.63689	2.29856(14^a^)	0.53534(5^a^)	1.83564(8^a^)

^a^: Number of standard samples; the number of samples for slave instrument with labels is 20 in TCR.

**Table 4:** RMSEP comparison of CTAI and other methods, RMSEP improvements and *p* values by the Wilcoxon signed rank test (α = 0.05). The m5spec was used as the master spectra, and the mp6spec was used as the secondary spectra for corn dataset. The protein content was chosen as the property for wheat dataset.

	MSC		TCR		CCA		SBC		PDS	
	*h*(%)	*p*	*h*(%)	*p*	*h*(%)	*p*	*h*(%)	*p*	*h*(%)	*p*
moisture	87.35	**4.3 × 10 ^−4^**	46	0.53	9.48	0.43	50.45	**0.01**	12.96	**0.04**
oil	93.31	**4.3 × 10 ^−4^**	42.12	**0.01**	27.98	0.32	1.52	0.23	13.28	0.46
protein	90.77	**4.3 × 10 ^−4^**	52.83	0.09	57.97	**0.03**	13.02	0.23	41.06	**0.01**
starch	82.02	**4.3 × 10 ^−4^**	56.52	0.23	59.49	0.83	6.09	**0.02**	56.04	0.75
B1*-B2	55.07	0.11	52.32	0.79	84.57	**5.3 × 10 ^−9^**	11.44	**2.6 × 10 ^−9^**	89.87	**9.2 × 10 ^−3^**
B1*-B3	71.64	**7.5 × 10 ^−10^**	6.55	0.11	38.3	**1.8 × 10 ^−5^**	13.97	**1 × 10 ^−5^**	48.92	**9.8 × 10 ^−5^**
B3*-B2	72.23	**3.1 × 10^−9^**	39.63	**4.6 × 10^−3^**	83.27	**0.02**	28.18	**7.5 × 10 ^−10^**	79.05	0.06
